# Calculation of likelihood ratios for inference of biological sex from human skeletal remains

**DOI:** 10.1016/j.fsisyn.2021.100202

**Published:** 2021-09-27

**Authors:** Geoffrey Stewart Morrison, Philip Weber, Nabanita Basu, Roberto Puch-Solis, Patrick S. Randolph-Quinney

**Affiliations:** a Forensic Data Science Laboratory, Computer Science Department & Aston Institute for Forensic Linguistics, Aston University, Birmingham, UK; b Forensic Evaluation Ltd, Birmingham, UK; c Leverhulme Research Centre for Forensic Science, University of Dundee, Dundee, UK; d Forensic Science Research Group, Department of Applied Sciences, Northumbria University, Newcastle upon Tyne, UK; e Department of Human Anatomy and Physiology, University of Johannesburg, South Africa

**Keywords:** Forensic inference and statistics, Forensic anthropology, Likelihood ratio, Sex assessment, Osteometry

## Abstract

It is common in forensic anthropology to draw inferences (e.g., inferences with respect to biological sex of human remains) using statistical models applied to anthropometric data. Commonly used models can output posterior probabilities, but a threshold is usually applied in order to obtain a classification. In the forensic-anthropology literature, there is some unease with this “fall-off-the-cliff” approach. Proposals have been made to exclude results that fall within a “zone of uncertainty”, e.g., if the posterior probability for “male” is greater than 0.95 then the remains are classified as male, and if the posterior probability for “male” is less than 0.05 then the remains are classified as female, but if the posterior probability for “male” is between 0.05 and 0.95 the remains are not classified as either male or female. In the present paper, we propose what we believe is a simpler solution that is in line with interpretation of evidence in other branches of forensic science: implementation of the likelihood-ratio framework using relevant data, quantitative measurements, and statistical models. Statistical models that can implement this approach are already widely used in forensic anthropology. All that is required are minor modifications in the way those models are used and a change in the way practitioners and researchers think about the meaning of the output of those models. We explain how to calculate likelihood ratios using osteometric data and linear discriminant analysis, quadratic discriminant analysis, and logistic regression models. We also explain how to empirically validate likelihood-ratio models.

## 1 Introduction

Forensic anthropology is the medico-legal application of biological anthropology. Forensic anthropologists apply to the analysis of human remains detailed knowledge of the development, the morphology, and the normal and abnormal variation of the human body. Analyses are conducted in order to assist legal-decision makers to make decisions with respect to identity of human remains [[1], [2], [3]]. Forensic anthropologists assist in the identification of individuals whose remains are severely decomposed, burned, disrupted, mutilated, or otherwise rendered difficult to recognize, particularly in cases where DNA evidence or odontological evidence are not available. Forensic anthropologists work on investigations related to unexplained natural deaths, accidents, homicide, war crimes, and genocide. They also increasingly work on disaster-victim identification, i.e., investigations related to mass fatality such as occur in building collapses, ship sinkings, and plane crashes.

Forensic anthropologists conduct evaluations with respect to chronological age, biological sex, living stature, and ancestry or population affinity. The analytical methods used can be divided into:

• morphoscopic, i.e., based on visual assessment of shape and size; and
• anthropometric/osteometric, i.e., based on instrumental measurements. The term “osteometric” applies to methods based on measurement of skeletal elements in particular.

Morphoscopic methods traditionally require considerable experience observing and understanding skeletal variation between individuals, populations, and age groups, and may be highly subjective in practice. Anthropometric methods are generally considered to be more objective, at least in the sense that intra- and inter-observer reliability is easier to assess. The most commonly used anthropometric measurements are point to point distances and angles. Some practitioners use a combination of morphoscopic and anthropometric methods.

It is common in forensic anthropology to draw inferences using statistical models applied to anthropometric data. A recently published book on the use of statistics and probability in forensic anthropology Obertová et al. [4], for instance, includes multiple chapters by different authors describing multiple statistical methods, including cluster analysis [5], logistic regression [6], and discriminant function analysis [7].

Use of classification models is common, and binary classification models have long been used to draw inferences with respect to biological sex, e.g., [[8], [9], [10], [11], [12], [13]]. Commonly used models such as linear discriminant analysis, quadratic discriminant analysis, and logistic regression can output posterior probabilities, but in the forensic-anthropology literature a threshold is usually applied in order to obtain a classification.^1^ For example, if the posterior probability for “male” is greater than 0.5 (or equivalently the posterior probability for “female” is less than 0.5) then the bone is classified as coming from a male, and if the posterior probability for “male” is less than 0.5 (or equivalently the posterior probability for “female” is greater than 0.5) then the bone is classified as coming from a female. In the forensic-anthropology literature, e.g., [[14], [15], [16]], there is evidence of some unease with this “fall-off-the-cliff” approach in which results with very different posterior probabilities, e.g., 0.51 and 0.99 are treated the same but results with very similar posterior probabilities, e.g., 0.49 and 0.51 are treated differently.

**Fig. 1:**
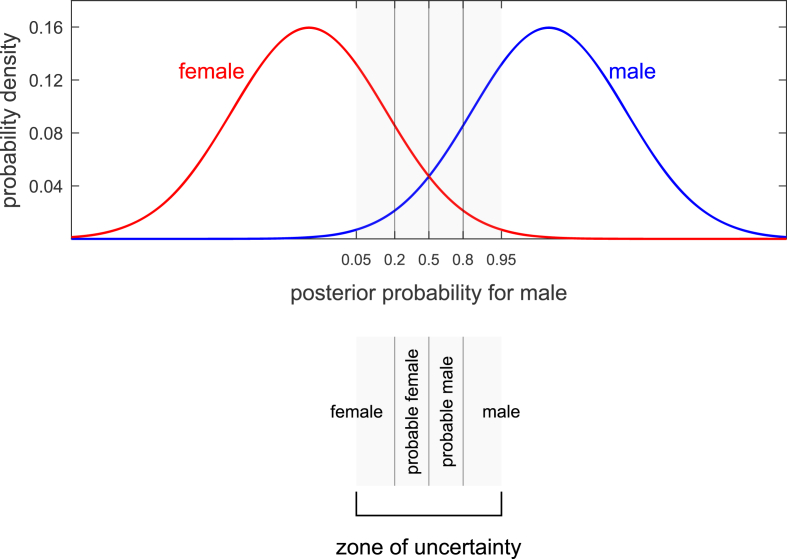
Example (based on humeral-head-diameter data from [18]) of a univariate linear discriminant analysis model showing multiple threshold values at different posterior probabilities for the hypothesis that the osteometric measurement comes from a male. In this example, the prior probabilities for “male” versus “female” are equal. Also shown are a “zone of uncertainty” between posterior probabilities of 0.05 and 0.95, and verbal expressions corresponding to the posterior probability ranges 0–0.2, 0.2–0.5, 0.5–0.8, and 0.8–1 (the latter proposed in [18]).

Galeta & Brůžek [7] reviews literature that expresses concern about a “zone of uncertainty”, see Fig. 1. In this “zone of uncertainty” the posterior probability is relatively close to 0.5, and the probability that a bone will be misclassified is relatively high. In order to attempt to avoid misclassification, a procedure is adopted whereby the bone is not classified unless the posterior probability is relatively far from 0.5, e.g., if the posterior probability for “male” is greater than 0.95 then the remains are classified as male, and if the posterior probability for “male” is less than 0.05 then the remains are classified as female, but if the posterior probability for “male” is between 0.05 and 0.95 the remains are not classified as either male or female. In this example, the “zone of uncertainty” is between posterior probabilities of 0.05 and 0.95. Galeta & Brůžek [7] states that “It is a conservative approach, but it brings a high confidence of sex estimation at both the individual and the population level.” The aim is to have a high correct-classification rate (a low classification-error rate) for the bones that are classified,^2^ but this comes at the cost of not classifying some bones and in fact not drawing any inference about the sex of the latter bones. Non-classification can occur in a high proportion, even the majority, of cases. Galeta & Brůžek [7] discusses trade-off between correct-classification rate and proportion of cases not classified.

Bartholdy et al. [18] propose reporting the correct-classification rate corresponding to the posterior-probability value calculated for the bone of interest. They propose either calculating the correct-classification rate at the exact posterior-probability value obtained, or precalculating the correct-classification rate for a number of preselected posterior-probability threshold values, e.g., 0.8, 0.9, 0.95, then, once the posterior-probability value for the bone of interest is obtained, selecting the relevant precalculated result, i.e., if the exact posterior-probability value obtained is between 0.8 and 0.9, report the correct-classification rate that was precalculated excluding test results with posterior-probability values between 0.2 and 0.8, if the exact posterior-probability value obtained is between 0.9 and 0.95, report the correct-classification rate that was precalculated excluding test results with posterior-probability values between 0.1 and 0.9, etc. Bartholdy et al. [18] also suggests that results could be reported as “female”, “probable female”, “probable male”, and “male” for posterior-probability ranges of, e.g., 0–0.2, 0.2–0.5, 0.5–0.8, and 0.8–1 respectively (see Fig. 1). Jerković et al. [17] propose the inverse solution of choosing a desired correct-classification rate and then finding the posterior-probability range that should be excluded in order to obtain this correct-classification rate.^3^

In the present paper, we propose what we believe is a simpler solution to the concerns expressed in the forensic-anthropology literature. We propose a move away from approaches in which the output is discretized into two or more bins, to an approach which makes direct use of continuously-valued output. Statistical models that can implement this approach are already widely used in forensic anthropology – all that is required to adopt this approach are minor modifications in the way those models are used and a change in the way practitioners and researchers think about the meaning of the output of the models. What we propose is implementation of the likelihood-ratio framework using relevant data, quantitative measurements, and statistical models.

We focus on explaining how to calculate likelihood ratios using linear discriminant analysis, quadratic discriminant analysis, and logistic regression models applied to osteometric data. For simplicity of exposition, we use data consisting of measurements made on a single skeletal element from each individual. The skeletal element we use is a humerus – humeri exhibit sexual dimorphism. The computer code for performing the calculations described in the present paper is provided at http://geoff-morrison.net/#LR_anthropology_2021. Parallel versions of the code are provided for Matlab, Python, and R.

## 2 Likelihood-ratio framework

Use of the likelihood-ratio framework is advocated by many who work in the area of forensic inference and statistics, e.g., Aitken et al. [19] with 31 authors/supporters, Morrison et al. [20] with 19 authors/supporters, and Morrison et al. [21] with 20 authors/supporters. Its use is also recommended in guidance documents issued by the following organizations:

• Association of Forensic Science Providers of the United Kingdom and of the Republic of Ireland (AFSP)^4^ in 2009 [22].
• Royal Statistical Society (RSS)^5^ in 2010 [23].
• European Network of Forensic Science Institutes (ENFSI)^6^ in 2015 [24].
• National Institute of Forensic Science of the Australia New Zealand Policing Advisory Agency (NIFS ANZPAA)^7^ in 2017 [25].
• American Statistical Association (ASA)^8^ in 2019 [26].
• Forensic Science Regulator for England & Wales (FSR)^9^ in 2021 [27].

Introductory texts on the likelihood-ratio framework include [[28], [29], [30], [31], [32], [33], [34], [35]]. Publications advocating or describing application of the likelihood-ratio framework in forensic anthropology include [[36], [37], [38], [39], [40], [41]].^10^

In the present paper, we do not attempt to provide a general introduction to the likelihood-ratio framework and arguments in favour of its use. Such information can be found in the references listed above. Instead, we focus on how to calculate likelihood ratios using the kinds of data and statistical models already familiar to practitioners and researchers in forensic anthropology. More complicated models can be used, and could potentially result in better performance, but for simplicity we focus on linear discriminant analysis, quadratic discriminant analysis, and logistic regression.^11^

For illustrative purposes, we use the humeral-measurement data from Bartholdy et al. [18]. The dataset contains measurements of maximum length, head diameter, and epicondylar breadth from the humeri of 36 males and 48 females. The dataset is small and the population does not reflect one that would be relevant for any modern forensic case, but it is a convenient dataset that will suffice to illustrate some statistical concepts. For univariate models we use the head-diameter measurements, and for bivariate models we use both head-diameter and epicondylar-breadth measurements.

The introductory literature on the likelihood-ratio framework tends to focus on what is often called “source attribution” or “individualization”, e.g., situations in which a legal-decision maker wants to decide whether the bone in question comes from a particular individual or from some other individual randomly selected from a specified relevant population. Here, we focus on a simpler “classification” problem with only two mutually-exclusive classes, e.g., a situation in which a legal-decision maker's task is to decide whether the skeletal element in question comes from a male or from a female from the specified relevant population.

## 3 Calculating a likelihood ratio using linear discriminant analysis

Traditionally in forensic anthropology, linear discriminant analysis is used to calculate a posterior probability to which a threshold is then applied to make a classification. When first developed, without the aid of modern computers, calculations for linear discriminant analysis were laborious. Linear discriminant functions were therefore used ([43], [44]). For a two-class problem, multivariate data could be transformed to values on a univariate linear discriminant function, and, assuming equal priors, each test datum could then be classified according to whether it was closer to the centroid of one class or the other. A higher prior probability for one class, and concomitantly lower prior probability for the other, would shift the threshold on the linear discriminant function further from the centroid of the first class and closer to the centroid of the second class. The calculation of the linear discriminant function was laborious, but thereafter classifying test data was easy as it did not require calculating the exact posterior probability for each new datum.

Using modern computers, the calculation of posterior probabilities (or of likelihoods) based on Gaussian distributions is trivial: all that is required is to enter training data into functions that calculate mean vectors and covariance matrices, then enter those statistics and the test data into functions that calculate probability densities. These functions are easily accessible in many programming languages and software packages. A posterior probability can be calculated as in Eq. (1), in which: $H_{\text{M}}$ is the hypothesis that the humerus comes from a male in the relevant population; $H_{\text{F}}$ is the hypothesis that the humerus comes from a female in the relevant population; $p\left(H_{\text{M}}|x_{\text{Q}}\right)$ is the posterior probability that the “male” hypothesis $H_{\text{M}}$ is true given the measurement vector $x_{\text{Q}}$ from the bone in question; f(x|μ,Σ) is the probability density (the likelihood) of a Gaussian model with mean vector μ and covariance matrix Σ evaluated at vector x; $\mu_{\text{M}}$ and $\mu_{\text{F}}$ are mean vectors calculated using a sample of data known to come from males in the relevant population and a sample of data known to come from females in the relevant population respectively; Σ is a covariance matrix calculated using data pooled from both the male and female samples^12^; $p\left(H_{\text{M}}\right)$ is the prior probability that the “male” hypothesis is true; and $p\left(H_{\text{F}}\right)$ is the prior probability that the “female” hypothesis $H_{\text{F}}$ is true.

(1) $$p\left(H_{\text{M}}|x_{\text{Q}}\right)=\frac{f\left(x_{\text{Q}}|\mu_{\text{M}},\Sigma \right)p\left(H_{\text{M}}\right)}{f\left(x_{\text{Q}}|\mu_{\text{M}},\Sigma \right)p\left(H_{\text{M}}\right)+f\left(x_{\text{Q}}|\mu_{\text{F}},\Sigma \right)p\left(H_{\text{F}}\right)}$$

Since $H_{\text{M}}$ and $H_{\text{F}}$ are mutually exclusive and exhaustive, $p\left(H_{\text{F}}\right)=1-p\left(H_{\text{M}}\right)$ and $p\left(H_{\text{F}}|x_{\text{Q}})=1-p(H_{\text{M}}|x_{\text{Q}}\right)$, and Eq. (1) can be rearranged to obtain Eq. (2), which is a version of the odds-form of Bayes’ Theorem.

(2) $$\frac{p(H_{\text{M}}|x_{\text{Q}})}{p(H_{\text{F}}|x_{\text{Q}})}=\frac{f(x_{\text{Q}}|\mu_{\text{M}},\Sigma )}{f(x_{\text{Q}}|\mu_{\text{F}},\Sigma )}\times \frac{p\left(H_{\text{M}}\right)}{p\left(H_{\text{F}}\right)}$$

posteriorodds=likelihoodratio×priorodds

In the odds-form of Bayes’ Theorem:

• the *prior odds* represent the legal-decision maker's belief as to the relative probability that the “male” hypothesis is true versus that the “female” hypothesis is true before they consider the forensic practitioner's statement of the strength of the evidence;
• the *likelihood ratio* is the forensic practitioner's statement of the strength of the evidence;
• and the *posterior odds* represent the legal-decision maker's belief as to the relative probability that the “male” hypothesis is true versus that the “female” hypothesis is true after they have considered the forensic practitioner's statement of the strength of the evidence.

The likelihood ratio therefore quantifies the amount by which, in light of the evidence, the legal-decision maker updates their belief with respect to the relative probabilities of the “male” and the “female” hypotheses. This assumes that the legal-decision maker is applying Bayes’ Theorem and using the likelihood ratio provided by the forensic practitioner. These assumptions are adopted in order to explain the meaning of a likelihood ratio, not to describe how a legal-decision maker actually acts or to advise how a legal-decision maker should act.

For the likelihood-ratio value to be meaningful, one must also be satisfied that the data used for training the statistical models (e.g., the data used for calculating the mean vectors and the covariance matrix) are reasonably representative of the relevant population for the case.

The prior odds could be based on an estimate of the ratio of males to females in the relevant population, but could also depend on other evidence already presented in the case that has influenced the legal-decision maker's belief with respect to the relative probabilities of the two hypotheses.

In the likelihood-ratio framework, the task of the forensic practitioner is to assess and present the value of the likelihood ratio. The likelihood-ratio value can, in theory, be any number in the range 0 to +∞ (the log-likelihood-ratio value can be any number in the range –∞ to +∞). The larger the number the greater the support it gives for the hypothesis in the numerator of the likelihood ratio (in this example, $H_{\text{M}}$), and the smaller the number the greater the support it gives for the hypothesis in the denominator of the likelihood ratio (in this example, $H_{\text{F}}$). If the likelihood-ratio value is 1 (the log-likelihood-ratio value is 0), it gives equal support for both hypotheses, and the posterior odds will be the same as the prior odds.

Assuming equal priors, $p\left(H_{\text{M}}\right)=p\left(H_{\text{F}}\right)$, hence prior odds $p\left(H_{\text{M}}\right)/p\left(H_{\text{F}}\right)=1$, a “zone of uncertainty” based on posterior probability for male between 0.05 and 0.95 would correspond to likelihood-ratio values in the range 0.05/0.95 to 0.95/0.05 (=1/19 to 19). Unlike an approach which does not draw any inference about the sex of bones with posterior probabilities within this “zone of uncertainty”, likelihood ratios provide meaningful information both outside and within this range, and they do not suffer from a “fall-off-the-cliff” effect. Likelihood-ratio values of 2, 10, or 1/15, for example, provide information that a legal-decision maker could logically use to update their beliefs, and likelihood-ratio values of 18.9 and 19.1 will not be presented to legal-decision makers as if they had very different meanings.

**Fig. 2:**
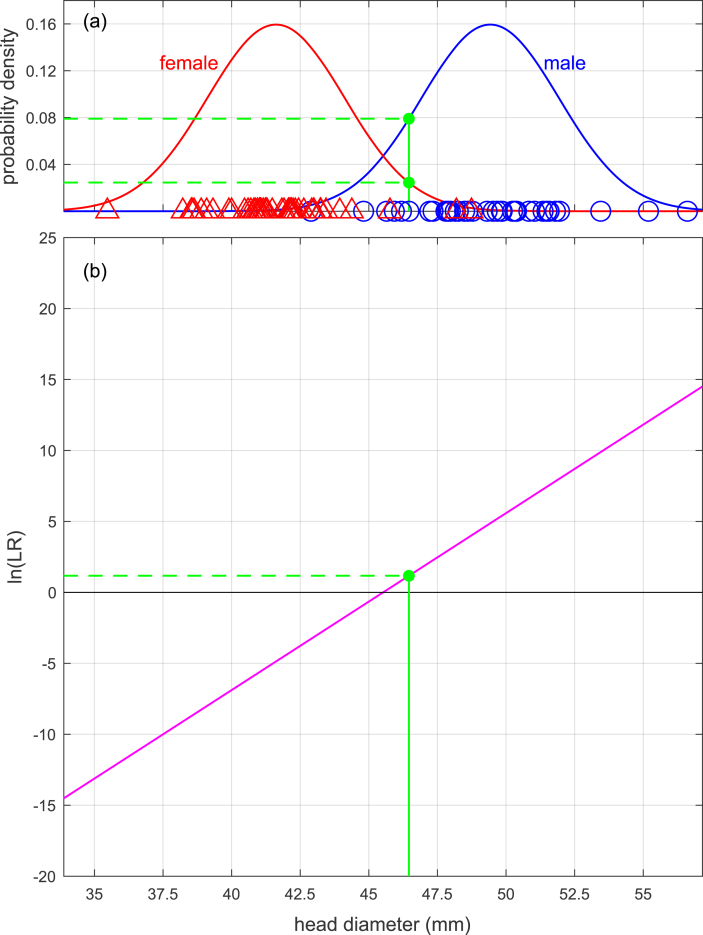
Example (based on humeral-head-diameter data from [18]) of calculation of likelihood ratio using a univariate linear discriminant analysis model. (a): Calculation based on probability-density functions. (b): Calculation based on a linear equation.

**Fig. 3:**
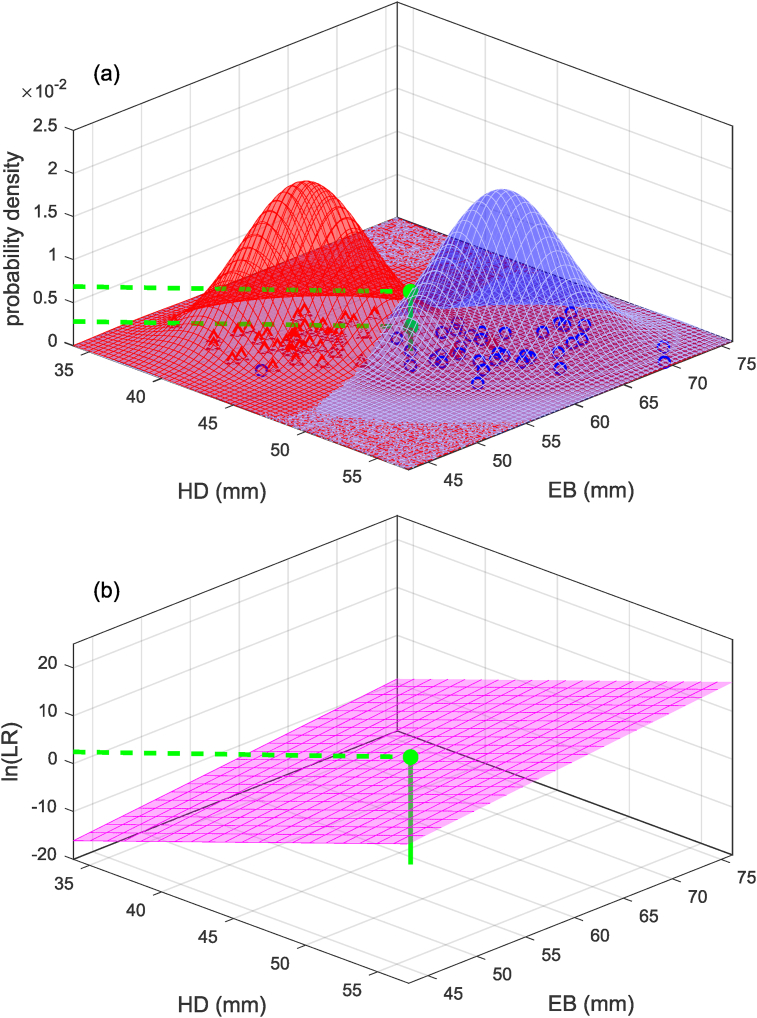
Example (based on humeral head-diameter, HD, and epicondylar-breadth, EB, data from [18]) of calculation of likelihood ratio using a bivariate linear discriminant analysis model. (a): Calculation based on probability-density functions. (b): Calculation based on a linear equation.

Eq. (3) shows a univariate example of the calculation of a likelihood ratio Λ(x), and Eq. (4) show a bivariate example of the calculation of a likelihood ratio Λ(x). Fig. 2(a) shows a graphical representation of Eq. (3) in which the likelihood ratio for measurement scalar x is the height of the “male” curve relative to the height of the “female” curve, and Fig. 3(a) shows a graphical representation of Eq. (4) in which the likelihood ratio for measurement vector x is the height of the “male” surface relative to the height of the “female” surface. The values inserted into the equations and used to plot the figures are taken from the Bartholdy et al. [18] dataset. One measurement (from a male) was selected and used as $x=x_{\text{Q}}$ in the univariate case and $x=x_{\text{Q}}$ in the bivariate case (hereinafter we drop the Q subscript), and the remainder of the data were used to calculate the values for $\mu_{\text{M}}$, $\mu_{\text{F}}$, σ, $\mu_{\text{M}}$, $\mu_{\text{F}}$, and Σ.

(3) $$\begin{array}{l} \text{Λ}\left(x\right)=\frac{f(x|\mu_{\text{M}},\sigma )}{f(x|\mu_{\text{F}},\sigma )}\\ =\frac{f(x=46.5\;|\;\mu_{\text{M}}=49.4,\;\sigma =2.50)}{f(x=46.5\;|\;\mu_{\text{F}}=41.6,\;\sigma =2.50)}\\ =\frac{0.0790}{0.0245}\\ =3.23 \end{array}$$

(4) $$\begin{array}{l} \text{Λ}\left(x\right)=\frac{f(x|\mu_{\text{M}},\Sigma )}{f(x|\mu_{\text{F}},\Sigma )}\;\\ =\frac{f(\left[\begin{array}{l} x_{1}\\ x_{2} \end{array}\right]|\left[\begin{array}{l} \mu_{\text{M},1}\\ \mu_{\text{M},2} \end{array}\right],\left[\begin{array}{ll} \sigma_{1,1} & \sigma_{1,2}\\ \sigma_{2,1} & \sigma_{2,2} \end{array}\right])}{f(\left[\begin{array}{l} x_{1}\\ x_{2} \end{array}\right]|\left[\begin{array}{l} \mu_{\text{F},1}\\ \mu_{\text{F},2} \end{array}\right],\left[\begin{array}{ll} \sigma_{1,1} & \sigma_{1,2}\\ \sigma_{2,1} & \sigma_{2,2} \end{array}\right])}\\ =\frac{f(\left[\begin{array}{l} 46.5\\ 59.0 \end{array}\right]|\left[\begin{array}{l} 49.4\\ 63.9 \end{array}\right],\left[\begin{array}{ll} 6.26 & 4.84\\ 4.84 & 15.8 \end{array}\right])}{f(\left[\begin{array}{l} 46.5\\ 59.0 \end{array}\right]|\left[\begin{array}{l} 41.6\\ 55.3 \end{array}\right],\left[\begin{array}{ll} 6.26 & 4.84\\ 4.84 & 15.8 \end{array}\right])}\\ =\frac{0.00687}{0.00282}\\ =2.44 \end{array}$$

Table 1 collects the example likelihood-ratio values calculated using the same measurement vector x and all the different models presented in the present paper.

**Table 1:** Example likelihood-ratio values calculated using the same example measurement vector and different univariate and bivariate models.

	Univariate (head diameter)	Bivariate (head diameter, epicondylar breadth)
Linear discriminant analysis	3.23	2.44
Logistic regression	2.26	1.91
Quadratic discriminant analysis	4.22	2.64

Before leaving linear discriminant analysis and moving on to logistic regression, in Eqs. (5), (6), (7) we show the derivation of the linear equation for the calculation of a likelihood ratio using linear discriminant analysis. For simplicity, we only show the derivation of the univariate equation: y=a+bx, in which y is the natural logarithm of the likelihood ratio, a is the intercept, b is the slope, and x is the head-diameter measurement made on the humerus.

(5) $$\begin{array}{l} y=\text{ln}\left(\text{Λ}\left(x\right)\right)=\text{ln}\left(\frac{f(x|\mu_{\text{M}},\sigma )}{f(x|\mu_{\text{F}},\sigma )}\right)\\ =\text{ln}\left(\frac{\frac{1}{\sigma \sqrt{2\pi }}e^{\frac{{\left(x-\mu_{\text{M}}\right)}^{2}}{-2\sigma^{2}}}}{\frac{1}{\sigma \sqrt{2\pi }}e^{\frac{{\left(x-\mu_{\text{F}}\right)}^{2}}{-2\sigma^{2}}}}\right)\\ =\text{ln}\left(e^{\frac{{\left(x-\mu_{\text{M}}\right)}^{2}-{\left(x-\mu_{\text{F}}\right)}^{2}}{-2\sigma^{2}}}\right)\\ =\frac{x^{2}+\mu_{\text{M}}^{2}-2x\mu_{\text{M}}-x^{2}-\mu_{\text{F}}^{2}+2x\mu_{\text{F}}}{-2\sigma^{2}}\\ =\frac{-\mu_{\text{M}}^{2}+2x\mu_{\text{M}}+\mu_{\text{F}}^{2}-2x\mu_{\text{F}}}{2\sigma^{2}}\\ =\frac{-\mu_{\text{M}}^{2}+\mu_{\text{F}}^{2}}{2\sigma^{2}}+\frac{\mu_{\text{M}}-\mu_{\text{F}}}{\sigma^{2}}x\\ =a+bx \end{array}$$

(6) $$b=\frac{\mu_{\text{M}}-\mu_{\text{F}}}{\sigma^{2}}$$

(7) $$a=\frac{-\mu_{\text{M}}^{2}+\mu_{\text{F}}^{2}}{2\sigma^{2}}=-b\frac{\mu_{\text{M}}+\mu_{\text{F}}}{2}$$

In Eqs. (8), (9)), we show a univariate example of the calculation of a likelihood ratio Λ(x) given the same values as previously used in Eq. (3). Note that the final result in Eq. (9) is the same as the final result in Eq. (3). The same example is graphically represented in Fig. 2(b). Note that the straight line in Fig. 2(b) could be constructed by sweeping a probe along the *x* axis of Fig. 2(a) and at each point calculating the natural logarithm of the height of the “male” curve relative to the height of the “female” curve.

(8) $$y=a+bx\;=\frac{-\mu_{\text{M}}^{2}+\mu_{\text{F}}^{2}}{2\sigma^{2}}+\frac{\mu_{\text{M}}-\mu_{\text{F}}}{\sigma^{2}}x\;=\frac{-49.4^{2}+41.6^{2}}{2\times 2.50^{2}}+\frac{49.4-41.6}{2.50^{2}}\times 46.5\;=-56.8+1.25\times 46.5\;=1.17$$

(9) $$\text{Λ}\left(x\right)=e^{y}=e^{1.17}=3.23$$

The bivariate example is graphically represented in Fig. 3(b). Note that the plane in Fig. 3(b) could be constructed by sweeping a probe around the $x_{1}$-$x_{2}$ plane of Fig. 3(a) and at each point calculating the natural logarithm of the height of the “male” surface relative to the height of the “female” surface. The multivariate equation in general would be: $y=\beta_{0}+\beta_{1}x_{1}+\beta_{2}x_{2}+\ldots +\beta_{m}x_{m}$, in which $\beta_{0}$ is the intercept and $\beta_{1},\ldots ,\;\beta_{m}$ are the slopes corresponding to the m dimensions of the data.

## 4 Calculating a likelihood ratio using logistic regression

Traditionally in forensic anthropology, logistic regression is used to calculate a posterior probability to which a threshold is then applied to make a classification. A posterior probability can be calculated as in Eq. (10), in which $\beta_{0}$ is an intercept and $\beta_{1},\ldots ,\;\beta_{m}$ are slopes corresponding to the m dimensions of the data. The values for $\beta_{0},\ldots ,\;\beta_{m}$ are calculated using an iterative algorithm. We do not describe the details of fitting logistic-regression models here, the interested reader is referred to texts such as [45] and [46]. For our calculations, we used the Newton iterative fitting algorithm with conjugate gradient ascent.

(10) $$p\left(H_{\text{M}}|x\right)=\frac{1}{1+e^{-\left(\beta_{0}+\beta_{1}x_{1}+\beta_{2}x_{2}+\ldots +\beta_{m}x_{m}\right)}}$$

Since $H_{\text{M}}$ and $H_{\text{F}}$ are mutually exclusive and exhaustive, $p\left(H_{\text{F}}|x)=1-p(H_{\text{M}}|x\right)$, and Eq. (10) can be rearranged to obtain Eq. (11). Eq. (11) gives the logged posterior odds, and this is the form in which the model is actually fitted.

(11) $$\text{ln}\left(\frac{p(H_{\text{M}}|x)}{p(H_{\text{F}}|x)}\right)=\beta_{0}+\beta_{1}x_{1}+\beta_{2}x_{2}+\ldots +\beta_{m}x_{m}$$

**Fig. 4:**
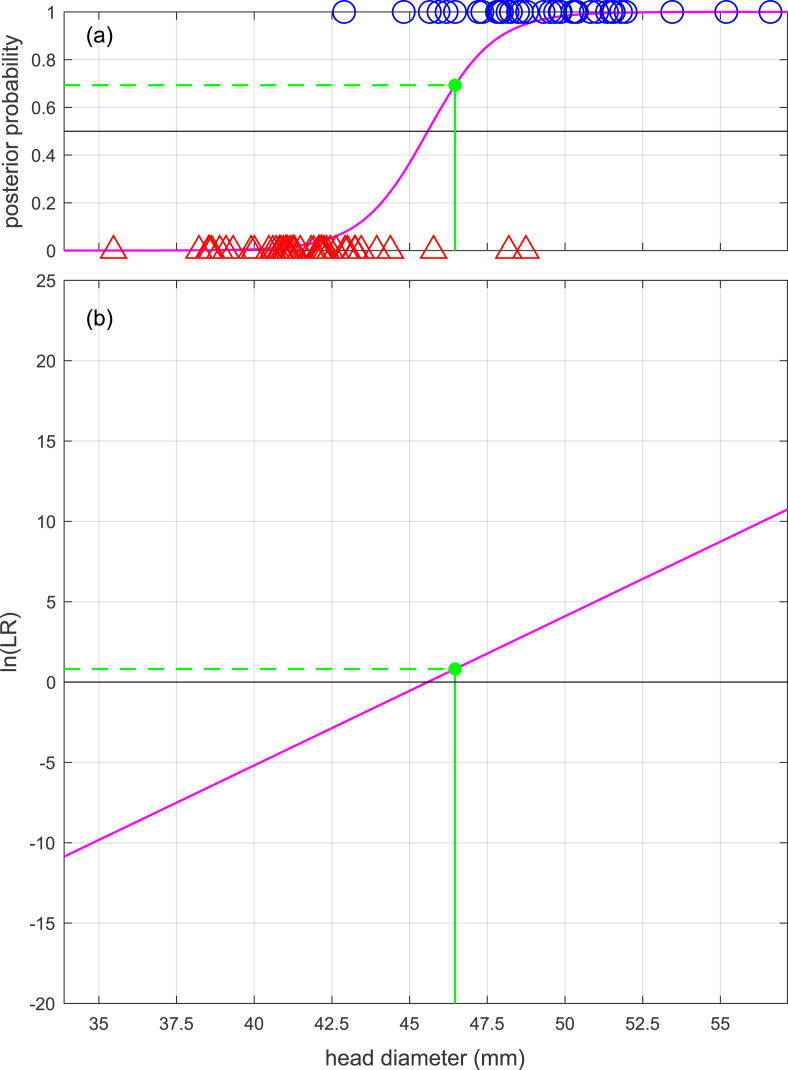
Example (based on humeral-head-diameter data from [18]) of calculation of likelihood ratio using a univariate logistic regression model. Compare Fig. 4(b) with Fig. 2(b).

**Fig. 5:**
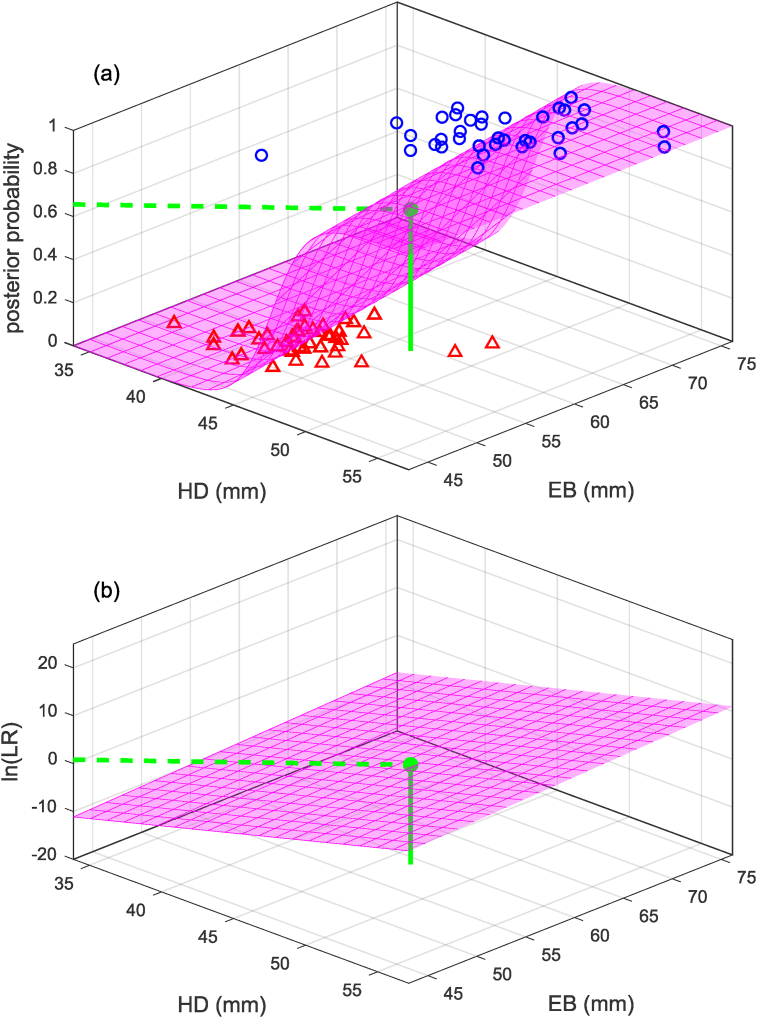
Example (based on humeral head-diameter, HD, and epicondylar-breadth, EB, data from [18]) of calculation of likelihood ratio using a bivariate logistic regression model. Compare Fig. 5(b) with Fig. 3(b).

In order to use logistic regression to calculate a likelihood ratio, the data points in the training data should be weighted such that the two classes have the same weight; hence, $p\left(H_{\text{M}}\right)=p\left(H_{\text{F}}\right)$, the prior odds $p\left(H_{\text{M}}\right)/p\left(H_{\text{F}}\right)=1$, and the posterior odds will equal the likelihood ratio (see Eq. (2)). Eqs. (12), (13), (14), (15) repeat the same examples as for linear discriminant analysis above but with the coefficients values (a and b, and $\beta_{0}$, $\beta_{1}$, $\beta_{2}$) obtained using logistic regression. For parallelism with the linear equation derived for linear discriminant analysis in the previous section, the univariate example uses a and b for the intercept and slope. Note that the values for a and b in Eq. (12) are not the same as those obtained using linear discriminant analysis in Eq. (8). Fig. 4, Fig. 5 show a graphical representation of the calculation of the likelihood ratio for the univariate and bivariate examples. Compare Fig. 4, Fig. 5 with Fig. 2, Fig. 3 respectively. In these examples, the slopes obtained using logistic regression are all shallower then the slopes obtained using linear discriminant analysis.

(12) ln(Λ(x))=y=a+bx=−42.3+0.928×46.5=0.815

(13) $$\text{Λ}\left(x\right)=e^{y}=e^{0.815}=2.26$$

(14) $$\text{ln}\left(\text{Λ}\left(x\right)\right)=y=\beta_{0}+\beta_{1}x_{1}+\beta_{2}x_{2}=-42.9+0.809\times 46.5+0.102\times 59.0=0.648$$

(15) $$\text{Λ}\left(x\right)=e^{y}=e^{0.648}=1.91$$

Logistic regression is a discriminative model, not a generative model – it does not actually calculate the ratio of two likelihoods – but under ideal circumstances it would give the same results as linear discriminant analysis ([47] §4.4.5).^13^ Because of its analogy with linear discriminant analysis, a generative model which actually calculates the ratio of two likelihoods, the output of logistic regression can be interpreted as a log likelihood ratio. Because it is not dependent on the assumptions of Gaussian distributions with the same covariance matrix, logistic regression is more robust than linear discriminant analysis when the data deviate from those assumptions. If the assumptions are met and the sample size is small; however, linear discriminant analysis will be less prone to overfit the training data.

## 5 Calculating a likelihood ratio using quadratic discriminant analysis

Quadratic discriminant analysis is the same as linear discriminant analysis, except that (in the present context) instead of using a single covariance matrix Σ calculated using data pooled from male and female samples, it uses two separate covariance matrices. $\Sigma_{\text{M}}$ is calculated using data sampled from males and $\Sigma_{\text{F}}$ is calculated using data sampled from females. Eq. (16) gives the quadratic-discriminant-analysis version of the odds-form of Bayes’ Theorem, cf. Eq. (2).

(16) $$\frac{p(H_{\text{M}}|x_{\text{Q}})}{p(H_{\text{F}}|x_{\text{Q}})}=\frac{f(x_{\text{Q}}|\mu_{\text{M}},\Sigma_{\text{M}})}{f(x_{\text{Q}}|\mu_{\text{F}},\Sigma_{\text{F}})}\times \frac{p\left(H_{\text{M}}\right)}{p\left(H_{\text{F}}\right)}$$

posteriorodds=likelihoodratio×priorodds

**Fig. 6:**
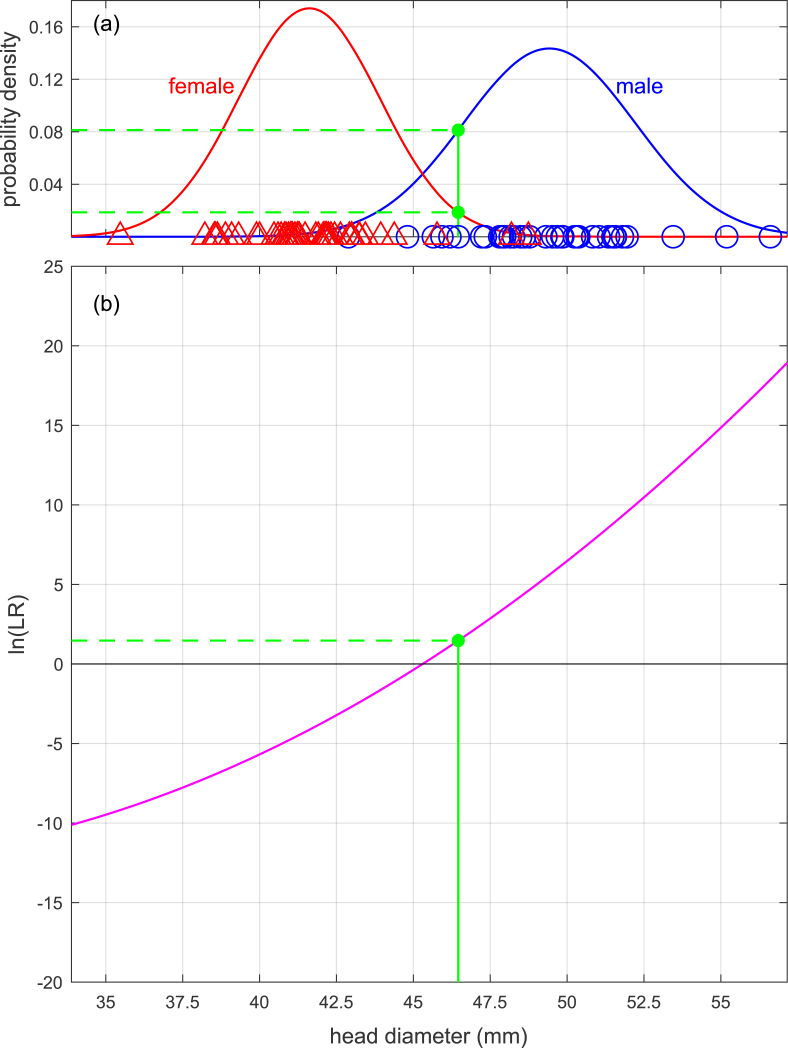
Example (based on humeral-head-diameter data from [18]) of calculation of likelihood ratio using a univariate quadratic discriminant analysis model.

**Fig. 7:**
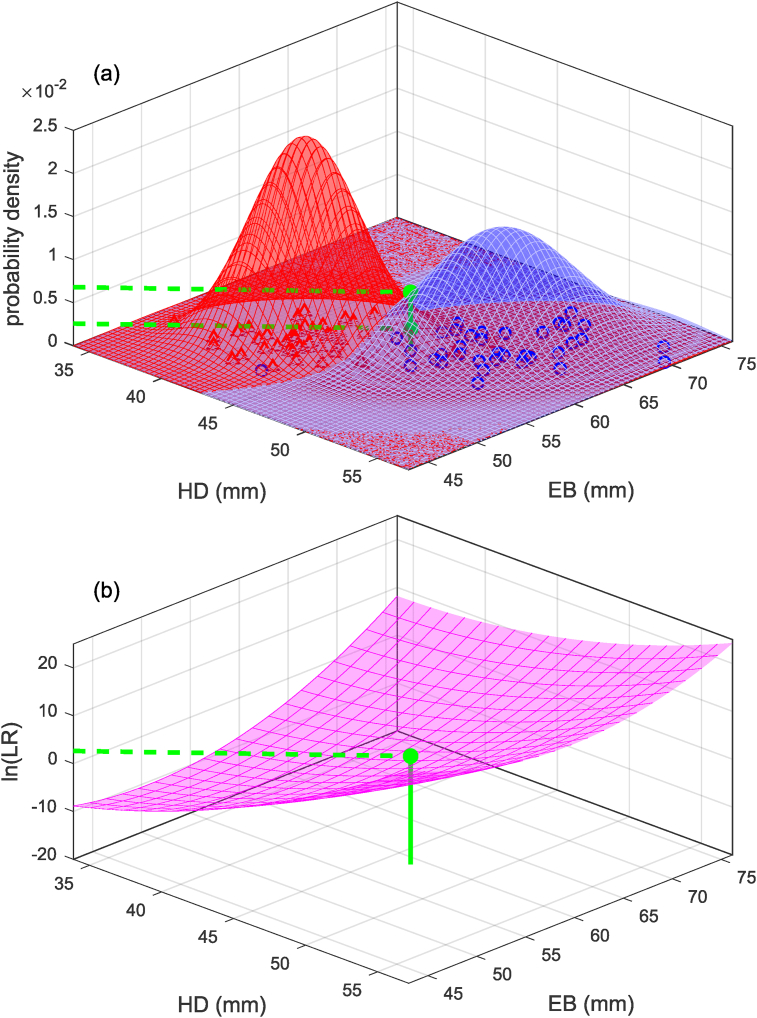
Example (based on humeral head-diameter, HD, and epicondylar-breadth, EB, data from [18]) of calculation of likelihood ratio using a bivariate quadratic discriminant analysis model.

Fig. 6 and Eq. (17) show the univariate example of the calculation of a likelihood ratio, and Fig. 7 and Eq. 18 show the bivariate example. Note that in Fig. 6, Fig. 7 the mapping functions between x and ln(Λ(x)) and between x and ln(Λ(x)) are not linear, they are a curve and a curved surface respectively.

(17) $$\text{Λ}\left(x\right)=\frac{f(x|\mu_{\text{M}},\sigma_{\text{M}})}{f(x|\mu_{\text{F}},\sigma_{\text{F}})}\;=\frac{f(x=46.5\;|\;\mu_{\text{M}}=49.4,\;\sigma_{\text{M}}=2.78)}{f(x=46.5\;|\;\mu_{\text{F}}=41.6,\;\sigma_{\text{F}}=2.31)}\;=\frac{0.0813}{0.0192}\;=4.22$$

(18) $$\text{Λ}\left(x\right)=\frac{f(x_{\text{Q}}|\mu_{\text{M}},\Sigma_{\text{M}})}{f(x_{\text{Q}}|\mu_{\text{F}},\Sigma_{\text{F}})}\;=\frac{f(\left[\begin{array}{l} x_{1}\\ x_{2} \end{array}\right]|\left[\begin{array}{l} \mu_{\text{M},1}\\ \mu_{\text{M},2} \end{array}\right],\left[\begin{array}{ll} \sigma_{\text{M},1,1} & \sigma_{\text{M},1,2}\\ \sigma_{\text{M},2,1} & \sigma_{\text{M},2,2} \end{array}\right])}{f(\left[\begin{array}{l} x_{1}\\ x_{2} \end{array}\right]|\left[\begin{array}{l} \mu_{\text{F},1}\\ \mu_{\text{F},2} \end{array}\right],\left[\begin{array}{ll} \sigma_{\text{F},1,1} & \sigma_{\text{F},1,2}\\ \sigma_{\text{F},2,1} & \sigma_{\text{F},2,2} \end{array}\right])}\;=\frac{f(\left[\begin{array}{l} 46.5\\ 59.0 \end{array}\right]|\left[\begin{array}{l} 49.4\\ 63.9 \end{array}\right],\left[\begin{array}{ll} 7.71 & 6.93\\ 6.93 & 23.2 \end{array}\right])}{f(\left[\begin{array}{l} 46.5\\ 59.0 \end{array}\right]|\left[\begin{array}{l} 41.6\\ 55.3 \end{array}\right],\left[\begin{array}{ll} 5.35 & 3.43\\ 3.43 & 10.7 \end{array}\right])}\;=\frac{0.00680}{0.00258}\;=2.64$$

## 6 Validation of likelihood-ratio models

The performance of a model is assessed by:

1. Taking data that represent the relevant population for the case, that reflect conditions of the case, and for which the true class of each datum is known (e.g., each measurement vector is made on a humerus known to be from a male or know to be from a female from the population of interest);
2. Inputting each measurement vector into the model;
3. Then comparing the output of the model in response to each input with the known truth about the class of the corresponding input.

The test data must be separate from the data used to train the model, otherwise the results will be overly optimistic with respect to how well the model will perform when applied to previously unseen data, e.g., the measurements made on the humerus of questioned biological sex in the case.

Typically in the forensic-anthropology literature, the results are summarized using correct-classification rate, i.e., the proportion of all inputs that were correctly classified.^14^ In the examples used in the present paper, the class of each input is either “male” or “female”. In a classification framework, the class of each output would be either “male” or “female”. If there is an imbalance in the number of “male” inputs and the number of “female” inputs in the validation data, the correct-classification rate can be separately calculated for each input class, then the mean over both classes calculated.

An alternative to correct-classification rate is classification-error rate, which is the proportion of inputs that were misclassified. This is equivalent to one minus the correct-classification rate.^15^ The classification-error rate, $E_{\mathrm{class}}$, with equal weighting for each class can be calculated as in Eq. (19), in which $N_{\text{M}}$ and $N_{\text{F}}$ are the number of inputs in the validation data known to be from males and the number of inputs in the validation data known to be from to be from females respectively, and $Y_{\text{M}}$ and $Y_{\text{F}}$ are classification outputs from the model in response to inputs known to be from males and inputs known to be from females respectively. In Eq. (19), a cost of 0 is assigned for a correct classification and a cost of 1 for an incorrect classification, the mean cost is calculated for inputs known to be from males and separately the mean cost is calculated for inputs known to be from females, then the mean of the latter two means is calculated. $E_{\mathrm{class}}$ is an average cost calculated over all the test data.

(19) $$E_{\mathrm{class}}=\frac{1}{2}\left(\frac{1}{N_{\text{M}}}\sum_{i}^{N_{\text{M}}}\left(\begin{array}{l} 0\;\text{if}\;Y_{{\text{M}}_{i}}=\text{M}\\ 1\;\text{if}\;Y_{{\text{M}}_{i}}=\text{F} \end{array}\right)+\frac{1}{N_{\text{F}}}\sum_{j}^{N_{\text{F}}}\left(\begin{array}{l} 0\;\text{if}\;Y_{{\text{F}}_{j}}=\text{F}\\ 1\;\text{if}\;Y_{{\text{F}}_{j}}=\text{M} \end{array}\right)\right)$$

$E_{\mathrm{class}}$ is a number between 0 and 1 inclusive. Lower $E_{\mathrm{class}}$ values indicate better performance, i.e., fewer misclassifications. The expected $E_{\mathrm{class}}$ value for a model whose output was random would be 0.5. A model with an $E_{\mathrm{class}}$ value greater then 0.5 would be performing worse than chance.

In the likelihood-ratio framework, the output of the model is not a classification but a continuously-valued likelihood-ratio value. In our examples, which have $H_{\text{M}}$ in the numerator and $H_{\text{F}}$ in the denominator, the higher the likelihood-ratio value the greater the support for $H_{\text{M}}$ relative to $H_{\text{F}}$ and the lower the likelihood-ratio value the greater the support for $H_{\text{F}}$ relative to $H_{\text{M}}$. If the input is from a male, the higher the likelihood-ratio value the greater the support for the correct hypothesis relative to the incorrect hypothesis. *Mutatis mutandis*, if the input is from a female, the lower the likelihood-ratio value the greater the support for the correct hypothesis relative to the incorrect hypothesis. Therefore, in order to assess the performance of a model that outputs likelihood ratios, we should not assign a cost of 0 or 1 based on classification, but rather a cost based on how good or how bad each likelihood-ratio values is:

• If we know the input was from a male we should assign a small cost value for a very large likelihood-ratio value, a larger cost value for a smaller likelihood-ratio value, and a much larger cost value for a very small likelihood-ratio value.
• *Mutatis mutandis*, if we know the input was from a female we should assign a small cost value for a very small likelihood-ratio value, a larger cost value for a larger likelihood-ratio value, and a much larger cost value for a very large likelihood-ratio value.

**Fig. 8:**
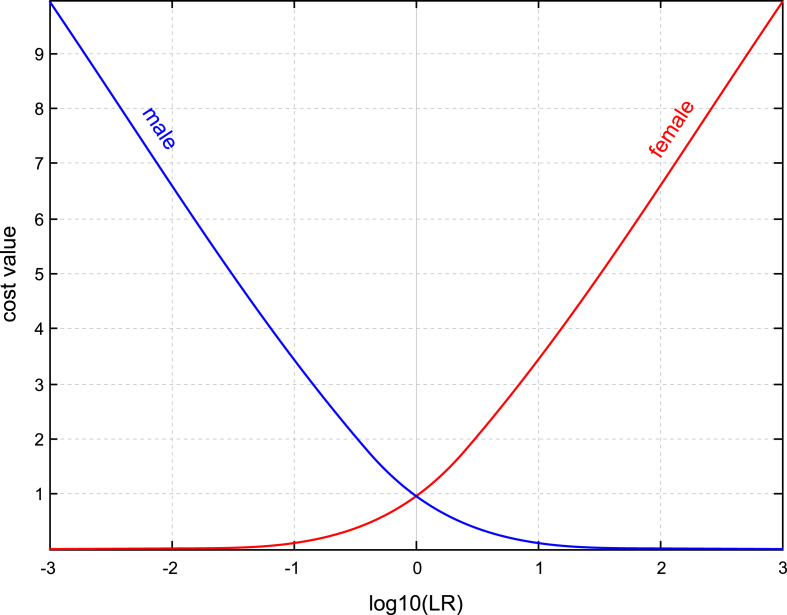
Cost functions within the leftmost summation and rightmost summation of Eq. (20).

A commonly used metric in the forensic-inference-and-statistics literature (and especially in the forensic-voice-comparison literature [21]) is the log-likelihood-ratio cost, $C_{\mathrm{llr}}$ [48], see Eq. (20), in which ${\text{Λ}}_{\text{M}}$ and ${\text{Λ}}_{\text{F}}$ are likelihood-ratio outputs from the model in response to inputs known to be from males and inputs known to be from females respectively. The functions within the leftmost summation and rightmost summation of Eq. (20) are plotted in Fig. 8.

(20) $$C_{\mathrm{llr}}=\frac{1}{2}\left(\frac{1}{N_{\text{M}}}\sum_{i}^{N_{\text{M}}}log_{2}\left(1+\frac{1}{{\text{Λ}}_{{\text{M}}_{i}}}\right)+\frac{1}{N_{\text{F}}}\sum_{j}^{N_{\text{F}}}log_{2}\left(1+{\text{Λ}}_{{\text{F}}_{j}}\right)\right)$$

$C_{\mathrm{llr}}$ is a number between 0 and +∞. Lower $C_{\mathrm{llr}}$ values indicate better performance. A model that always output a likelihood ratio of 1 irrespective of the input would give no useful information: the posterior odds would always be the same as the prior odds. A model that gave no useful information would have a $C_{\mathrm{llr}}$ value of 1. Models that are miscalibrated can output likelihood ratios substantially larger than 1, but their performance can be improved by calibrating the system (see [49] for an introduction to this topic). Well calibrated systems will have $C_{\mathrm{llr}}$ values in the range 0 to ∼1.

Returning to our univariate and bivariate examples, we validate the previously described models using leave-one-out cross validation, in which one measurement vector is held out, the remainder of the vectors are used to train the model, and the likelihood-ratio value is then calculated for the held-out vector. This is then repeated holding out each measurement vector in turn. This makes best use of the limited amount of data available while still avoiding training and testing on the same data. The resulting $C_{\mathrm{llr}}$ values are given in Table 2.

**Table 2:** values for different likelihood-ratio models applied to data from [18].

	Univariate (head diameter)	Bivariate (head diameter, epicondylar breadth)
Linear discriminant analysis	0.300	0.341
Logistic regression	0.306	0.349
Quadratic discriminant analysis	0.321	0.339

Based on the $C_{\mathrm{llr}}$ values in Table 2, the univariate models performed better than the bivariate models.^16^ The simpler univariate linear models (linear discriminant analysis and logistic regression) also performed a little better than the more complex univariate quadratic discriminant analysis.

**Fig. 9:**
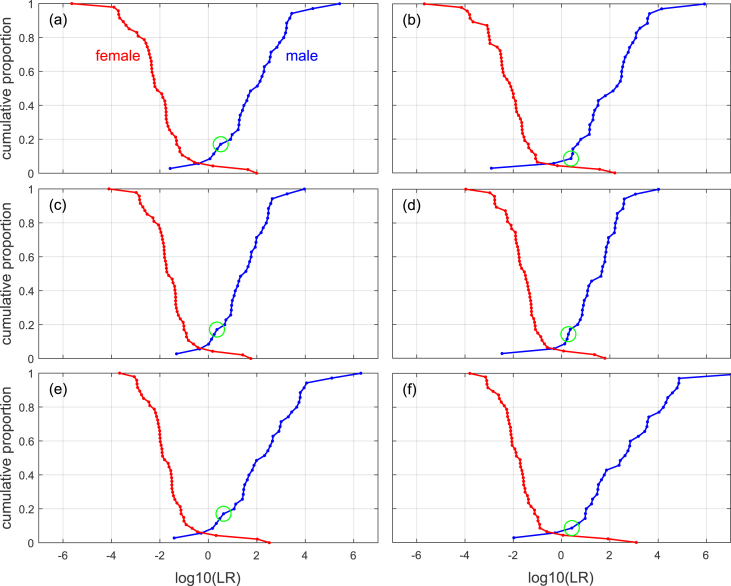
Tippett plots for different likelihood-ratio models applied to data from [18]. (a) Univariate linear discriminant analysis. (b) Bivariate linear discriminant analysis. (c) Univariate logistic regression. (d) Bivariate logistic regression. (e) Univariate quadratic discriminant analysis. (f) Bivariate quadratic discriminant analysis. In each panel, the dot in the middle of the circle corresponds to the result from the example feature vector.

A graphical representation of likelihood-ratio validation results commonly used in the forensic-inference-and-statistics literature (and especially in the forensic-voice-comparison literature [21]) is a Tippett plot [50]. Tippett plots for the previously described likelihood-ratio models are given in Fig. 9. The likelihood-ratio value corresponding to each measurement vector is plotted as a dot, and straight lines are drawn between adjacent dots. In our examples, a Tippett plot displays the empirical cumulative distribution of all the likelihood-ratio values resulting from test data known to be from males, and the empirical cumulative distribution of all the likelihood-ratio values resulting from test data known to be from females. The empirical cumulative distributions are plotted so that for the curve rising to the right the value on the *y* axis is the proportion of male inputs resulting in likelihood-ratio values equal to or less than the value on the *x* axis, and for the curve rising to the left the value on the *y* axis is the proportion of female inputs resulting in likelihood-ratio values equal to or greater than the value on the *x* axis.

In general, the better the performance of the system that generated the likelihood-ratio results, the greater the separation between the “male” and “female” curves on the Tippett plots, and, concomitantly, the shallower the slopes of the curves. Given this, the results from quadratic discriminant analysis (shown in the bottom panels of Fig. 9) may appear to be better than the results from linear models (linear discriminant analysis and logistic regression shown in the top and middle panels), but the results from quadratic discriminant analysis also include some large-magnitude positive log-likelihood-ratio values for bones known to be from females. The results from the bivariate models (shown in the panels on the right) also include some large-magnitude positive log-likelihood-ratio values for bones known to be from females, and, in addition, some large-magnitude negative log-likelihood-ratio values for bones known to be from males. The extent of these likelihood-ratio results supporting contrary-to-fact hypotheses is less for the univariate linear models: univariate linear discriminant analysis and univariate logistic regression (shown in panels (a) and (c)). As already indicated by the $C_{\mathrm{llr}}$ values, the best results were obtained for the univariate linear models.

All models provide useful information, $C_{\mathrm{llr}}$ is substantially less than 1, and appear to give reasonably well-calibrated output – the curves in the Tippett plots cross relatively close to ln(*LR*) = 0. For more complex models in which larger numbers of parameter values need to be estimated, it is usually necessary to calibrate their output using an explicit calibration model, see [51], [21], and [52].

Some of the models output likelihood-ratio values into the tens of thousands and even into the millions. These numbers are difficult to justify given the small sample sizes. To avoid complicating the present paper we do not address this issue here, but direct the interested reader to some solutions explored in [53].

Considering both $C_{\mathrm{llr}}$ and Tippett plots and the discussion above, given the Bartholdy et al. [18] dataset, the univariate logistic regression model appears to have resulted in the best performance. Note that it did not give the “best” results for the example feature vector (it did not give the largest likelihood-ratio value for this male feature vector), but it gave the best results averaged over all feature vectors. Given the small dataset, its lack of relevance for any modern forensic context, and the fact that the epicondylar-breadth data violate the assumptions of all the models tested, one should not draw any generalizations from any of the particular results presented here.

For other descriptions of both $C_{\mathrm{llr}}$ and Tippett plots see [[54], [55], [56], [57]] and [21].

## 7 Conclusion

Use of the likelihood-ratio framework for evaluation of forensic evidence is advocated by many who work in the area of forensic inference and statistics, and in guidance documents issued by prominent organizations. So far, there has been little use of the likelihood-ratio framework in forensic anthropology, but, with respect to adoption of the likelihood-ratio framework, forensic anthropology has advantages over some other branches of forensic science: it is a branch of forensic science in which it is already common to draw inferences on the basis of relevant data, quantitative measurements, and statistical models. In the present paper, we explained how to calculate likelihood ratios using anthropometric data, and statistical models that are already commonly used in forensic anthropology: linear discriminant analysis, quadratic discriminant analysis, and logistic regression. We also explained how to empirically validate likelihood-ratio models. We hope that this will contribute to greater understanding and wider adoption of the likelihood-ratio framework in forensic-anthropology research and practice.

## Disclaimer

All opinions expressed in the present paper are those of the authors, and, unless explicitly stated otherwise, should not be construed as representing the policies or positions of any organizations with which the authors are associated.

## Author contributions

Geoffrey Stewart Morrison: Conceptualization, Writing – original draft, Writing – review & editing, Funding acquisition. Philip Weber: Investigation, Software, Visualization, Writing – review & editing. Nabanita Basu: Investigation, Software, Writing – review & editing. Roberto Puch-Solis: Software, Writing – review & editing. Patrick S. Randolph-Quinney: Writing – review & editing.

## Declaration of competing interest

The authors declare that they have no known competing financial interests or personal relationships that could have appeared to influence the work reported in this paper.

## Footnotes

1 In the forensic-anthropology literature, the term “sectioning point” is often used rather than “threshold”.

2 Jerković et al. [17] claims that a 95% correct-classification rate “is the minimal level set by modern forensic and legal standards”. We traced the publications that Jerković et al. [17] cited in support of this claim and the publications cited in those publications, but could find no support for the claim that this is a legal requirement. Nor could we find any evidence that it is a requirement of any standard on forensic science developed by a national or international standards-development organization.

3 Note that if the data used for training and testing the statistical models were sampled from the same populations and the population distributions conformed to the assumptions of the models, then the expected value of the correct-classification rate would be predictable from the posterior-probability threshold and vice versa. If the posterior-probability threshold were τ, i.e., only data with posterior probabilities for male $p\left(H_{\text{M}}\right)\geq \tau$ and $p\left(H_{\text{M}}\right)\leq \left(1-\tau \right)$, or equivalently $p\left(H_{\text{F}}\right)\geq \tau$, were used for calculating the correct-classification rate κ, then the expected correct-classification rate would be $\kappa =\left(p\left(p\left(H_{\text{M}}\right)\geq \tau \right)\;+p\left(p\left(H_{\text{F}}\right)\geq \tau \right)\right)/2=\left(\tau +\tau \right)/2=\tau$, e.g., if τ=0.95 then the expected value for κ=0.95. Despite the difference in name, “posterior-probability threshold” versus “correct-classification rate”, τ and κ represent the same underlying concept, with τ being the predicted value and κ being the empirically derived value. In practice τ and κ would usually differ because of violations of model assumptions, model overfitting, and/or sampling variability. With respect to sampling variability, keeping τ fixed but changing the sample used for training or the sample used for testing would usually result in a different value for κ. Separate sets of training and test data are used to assess the extent to which the model is useful.

10 The likelihood-ratio framework for evaluation of forensic evidence should not be confused with likelihood-ratio tests used to assess difference in goodness of fit between competing models. Konigsberg et al. [42], for example, makes use of likelihood-ratio tests. Other references to likelihood ratios in that paper, e.g., “Taken as an evidentiary problem and assuming equal priors for male as for female within the population at large, the LR from the quadratic discriminant function is 1.997. This is found by calculating the [multivariate normal] density for obtaining ‘Mr. Johnson's’ measurements from the males and from the females …, averaging these densities across the two sexes, and dividing the male density by this average.” (p. 80), are not likelihood ratios as understood in the likelihood-ratio framework. As defined in the quote, they are twice the posterior probability. The definition in the quote is equivalent to our Eq. (1) multiplied by two and assuming equal priors. The likelihood ratio corresponding to the value stated in the quote would actually be 666.

11 Discriminant analysis assumes that the data from each class have Gaussian distributions, and linear discriminant analysis further assumes that the distributions from all classes have the same variance (in the univariate case) or the same covariance matrix (in the multivariate case). Histogram plots of the Bartholdy et al. [18] data reveal that these assumptions do not hold for epicondylar-breadth measurements: the female data appear to have a positive skew and the male data appear to be bimodal. Logistic regression is more robust to violations of these assumptions, but will not be robust to the bimodal distribution of the male data. Exploratory analysis of the data therefore suggest that none of linear discriminant analysis, quadratic discriminant analysis, or logistic regression are appropriate. Our purpose here, however, is simply to illustrate how to use these models, that are common in forensic anthropology, to calculate likelihood ratios. Whether these are good models to apply to these data, how well they perform when applied to these data, and the likelihood-ratio values that they output when applied to these data are not actually of concern. Use of linear discriminant analysis and logistic regression in the present paper also allows for direct comparison with their use in Bartholdy et al. [18] with the same dataset.

12 We used the formula for the unbiased estimate of the covariance matrix, i.e., dividing by 1−n rather then by n (where n is the number of data point used to calculate the covariance matrix). We gave equal weight to each data point, i.e., we subtracted the class mean from the data in each class, pooled the data, and then calculated the covariance matrix.

13 A generative model is a model that estimates a probability density for the measurements.

14 In the forensic-anthropology literature, correct-classification rate is usually expressed as a percentage. In the present paper, we express it as a proportion.

15 If classification-error rate and correct-classification rate are expressed as a percentages, the classification-error rate is 100 minus the correct-classification rate.

16 As mentioned in note 11, the epicondylar-breadth data violates the assumptions of all the models tested. Epicondylar breadth and head diameter were also highly correlated (Pearson's linear correlation coefficient ρ=0.794). There may have been little additional useful information that the bivariate models could have exploited compared to their univariate counterparts, especially given the sampling variability associated with the small sample sizes. Univariate models based on epicondylar breadth had $C_{\text{llr}}$ values in the range 0.5–0.6.

## Footnotes

4 http://www.afsp.org.uk/.

5 https://rss.org.uk/.

6 https://enfsi.eu/.

7 https://www.anzpaa.org.au/forensic-science/nifs-home/.

8 https://www.amstat.org/.

9 https://www.gov.uk/government/organisations/forensic-science-regulator/.
